# Supplementary material for: Comparative Analysis of Developmental Transcriptome Maps of Arabidopsis thaliana and Solanum lycopersicum
Source: Genes (Basel). 2019 Jan 15;10(1):50. doi: 10.3390/genes10010050 (PMC6356586; doi:10.3390/genes10010050)
Supplement: Supplementary file 1 [file genes-10-00050-s001.zip › genes-410128-supplementary_final/genes-410128-supplementary_figures.rtf]

Genes 2019, 10, x; doi: FOR PEER REVIEW 	www.mdpi.com/journal/genes


Supplementary Figure S1. Hierarchical Clustering Tree. All samples were clustered based on Pearson r2. For each sample biological replicates are grouped together. The tree reflects general anatomy of the plant and has a similar structure to Arabidopsis clustering tree (Klepikova et al., 2016, Supplementary Figure S1).


Supplementary Figure S2. The number of genes expressed in samples. For each sample gene was considered as expressed if in both biological replicates it has 5 and more normalized TGR (weak threshold, green) or 15 and more normalized TGR (strong threshold, purple). The difference between sample with highest and lowest number of expressed genes was 3,597 and 3,216 for weak and strong thresholds, respectively, which was slightly lower than for A. thaliana – 4,088 (Klepikova et al., 2016, Supplementary Table S6).


Supplementary Figure S3. Distribution of genes by number of samples in which each gene was expressed. For each sample gene was considered as expressed if in both biological replicates it has 5 and more normalized TGR (weak threshold, green) or 16 and more normalized TGR (strong threshold, purple). The distribution with peaks at highest and lowest number of samples is similar to such distribution of A. thaliana transcriptome map (Klepikova et al., 2016, Figure 2a).


Supplementary Figure S4. The distribution of gene expression level on number of samples in which a gene is expressed for minimum, mean, median and maximum expression levels of each gene. As well as in case of A. thaliana (Klepikova et al., 2016, Figure 2b) widely expressed genes have higher expression level. Median of expression across genes for each number of samples is marked by a red dot.


Supplementary Figure S5. Distribution of Shannon entropy (H). Shannon entropy is a measure of expression width, where high level of H corresponds to ubiquitously expressed genes and low value to tissue-specific expression. As for A. thaliana, the Shannon entropy distribution (Klepikova et. al, 2016, Figure 3b) is shifted to the right side and has a peak of genes with low H, though less pronounced.


Supplementary Figure S6. Distribution of Shannon entropy (H) in tomato and Arabidopsis. Shannon entropy is a measure of expression width, where a high level of H corresponds to ubiquitously expressed genes and low value to tissue-specific expression. The distributions of H are quite similar for both species, with differences at lowest and highest H.


Supplementary Figure S7. Distribution of DE Score. DE score is the number of pairwise comparisons in which a gene is differentially expressed. In comparison with A. thaliana DE score distribution (Klepikova et al., 2016, Figure 3a), the histogram is shifted to the left and descents rapidly on the right side. Such distribution shape is caused by the lower number of samples in S. lycopersicum transcriptome map and the absence of samples with highly similar expression profiles.


Supplementary Figure S8. Distribution of Z-score for selected samples. The Z-score represents the difference between gene expression in a sample and the mean expression of the gene across all samples divided by the standard deviation of gene expression. For almost all comparable samples of Arabidopsis and Solanum, the distributions of Z-score were highly similar (Klepikova et al., 2016, Figure 2c).


Supplementary Figure S9. Mapping of reads on FRUIT dataset. The histogram shows percent of reads mapped uniquely on annotated genes. On the y-axis number of samples, on the x-axis % of uniquely mapped reads.
